# Gene expression profile of CD8 T cells from the responder and non-responder mice following immunotherapy treatment for prostate cancer

**DOI:** 10.1186/2051-1426-1-S1-P55

**Published:** 2013-11-07

**Authors:** Seema Dubey, Sumedha Gunewardena, Peter Van Veldhuizen, J Brantley Thrasher, Dev Karan

**Affiliations:** 1 Urology, The University of Kansas Medical Center, Kansas City, KS, USA; 2 Molecular and Integrative Physiology, The University of Kansas Medical Center, Kansas City, KS, USA; 3 Internal Medicine, The University of Kansas Medical Center, Kansas City, KS, USA

## 

Immunotherapy represents a novel treatment approach for cancer; however, a longer maturation time and the lack of immunological markers are major obstacles in the development and characterization of immunological therapies. This study aims to identify gene expression profile on CD8+ T cells between the responder (completely regressed tumors) and non-responder (progressively growing tumors) groups of mice helpful in predicting the outcome of immunotherapy treatment. Using a preclinical mouse model for prostate cancer, subcutaneous tumors were established and the mice were treated with Ad-PSA+PSCA bivalent vaccine. Splenic CD8+ T cells purified from the responder and non-responder groups of mice were subjected to Affymetrix microarray to obtain gene expression profile. Pooling the data sets from two independent experiments revealed an up-regulation of 85 genes (>2.0 fold; p =2.7e-63) and down-regulation of 1768 gene (>2.0 fold; p =1.0e-63) in CD8+ T cells from the non-responder mice. Gene network analysis showed a coordinated expression of genes implicated in cell-mediated immune response, cell-to-cell signaling and interaction, and immune cell trafficking. The mRNA expression levels for the selected transcripts were verified using semi-quantitative RT-PCR method suggesting a panel of genes specific to CD8+ T cells differentially expressed between the responder and non-responder mice. Further studies are warranted to determine the potential of these identified genes as immunological biomarker to predict immunotherapy response.

